# The correlated expression of COX-2 and keratin 15 in radicular cysts

**DOI:** 10.4317/jced.59443

**Published:** 2022-04-01

**Authors:** Mohammed-Amjed Alsaegh, Maher Al Shayeb, Sudhir-Rama Varma, Alaa-Muayad Altaie, Shengrong Zhu

**Affiliations:** 1 Department of Oral and Craniofacial Health Sciences, College of Dental Medicine, University of Sharjah. Sharjah, UAE; 2 Department of Oral and Maxillofacial Surgery, Tongji Hospital of Tongji Medical College, Huazhong University of Science and Technology. Wuhan, Hubei 430030, P.R. China; 3 Department of Clinical Sciences, College of Dentistry, Ajman University. Ajman, UAE; 4 Sharjah Institute for Medical Research, College of Medicine, University of Sharjah. Sharjah, UAE

## Abstract

**Background:**

The expression of cyclooxygenase-2 (COX-2) and Keratin-15 (K15) in radicular cysts (RCs) is poorly understood. Identifying the expression of these two markers may modify the current treatment of RC. The objective of this study was to evaluate the expression of COX-2 and its relationship to K15 expression in the odontogenic epithelial cells of the RC.

**Material and Methods:**

A total of 18 RCs were immunohistochemically analyzed for COX-2 and K15 expression. The cellular inflammatory reaction in the cyst wall was also assessed by measuring the percentage of inflammatory cells to the total number of cells.

**Results:**

COX-2 expression in the odontogenic epithelium of RC was absent in 11.1 % (n=2), mild in 27.8 % (n=5), moderate in 22.2% (n=4) and strong in 38.9% (n=7). Meanwhile, K15 expression was absent in 27.8% (n=5), mild in 16.7% (n=3), moderate in 44.4% (n=8), and strong in 11.1% (n=2) of the cases. The inflammatory infiltrate was mild in 2 cases (11.1%), moderate in 6 cases (33.3%), and high in 10 cases (55.6%). Spearman’s correlation test revealed significant correlation (rho= .533; *p*= .023) between COX-2 and K15 expression in the odontogenic epithelium of RC. However, no correlation was noted between inflammation and expression of COX-2 (rho= 0.248, *p*=.321) or K15 (rho= -0.162, *p*= .520).

**Conclusions:**

There is high and correlated expression of COX-2 and K15 in the odontogenic epithelium of RC. COX-2 could therefore be involved in epithelial cell differentiation of the cyst. Additionally, the expression of K15 in RC may be an indicator of epithelial cell differentiation.

** Key words:**Cyclooxygenase, COX-2, Keratin-15, K15, Radicular cyst.

## Introduction

Radicular cyst (RC) is an odontogenic inflammatory cyst, usually asymptomatic unless it becomes infected. It is an epithelium-lined cavity that is surrounded by a fibrous capsule and it is believed to evolve from inflamed granulation tissue in which the epithelial rests are stimulated by the inflammatory process (1).

Cyclooxygenase (COX) is the enzyme that produces prostanoids from arachidonic acid. Prostanoids include prostacyclins, prostaglandins, and thromboxanes. Two primary COX isoforms have been identified that are encoded by different genes. These are COX-1 and COX-2. While COX-1 is expressed constitutively as a ‘housekeeping’ enzyme, COX-2 can be either inducible or constitutive (2). In comparison to COX-1, COX-2 has a rapid expression but also a rapid turnover in response to appropriate stimuli. COX-2 expression is regulated by different cytokines and growth factors such as tumor necrosis factor alpha, interleukin 1 beta, or interleukin 6 (3), whereas anti-inflammatory cytokines, glucocorticoids and non-steroidal anti-inflammatory drugs (NSAIDs) repress COX-2 expression. It is believed that prostaglandin E2 stimulates osteoclastic bone resorption due to its effects as a proinflammatory mediator, vasodilator and inducer of capillary permeability. (4). Little is known about COX-2 expression in odontogenic cysts and tumors. COX-2 expression was investigated in periapical granuloma and periodontitis (5,6), RC (5-7), odontogenic keratocyst (8,9), dentigerous cyst, and ameloblastoma (10).

Keratins, before they were referred to as cytokeratins, form a complex network of intermediate filaments that extend from the nucleus to the periphery of the cell. These filaments insert into cellular junctions, such as hemidesmosomes and desmosomes. There are at least 20 different keratins in human tissues. Keratins are categorized into type I, which are smaller, more acidic polypeptides involving keratins 9-20 and type II, which are large and basic-neutral in charge polypeptides including keratins 1-8 (11). Keratins are expressed in pairs where a specific neutral keratin dimerizes with its acidic partner forming a cytoskeleton that maintains the shape and integrity of the epithelium. However, K15 lacks a type II partner, but instead it shares keratin-5 together with keratin-14. Therefore, in the absence of keratin-14, K15 makes a keratin filament network with K5 (12). Keratins are present in dental organs and therefore, in odontogenic cysts and tumors. The type and pattern of keratin expression were used to identify a cell and its different stages of differentiation (13). Providing the cytoskeleton and mechanical integrity of the epithelium is the main function of keratins. However, different keratins have various roles in the intracellular signaling pathways like in apoptosis, protection from stress, and wound healing (14). Meanwhile, the specific function of K15 in normal and pathological tissues is not fully understood (15). Generally, keratins have been used as fundamental markers for differentiation of epithelial cells. Thus, studies were conducted to evaluate the role of keratins in pathogenesis, diagnosis, and treatment protocols for various epithelial lesions (13). Different keratin expressions in RC were studied before (13). However, knowledge is little about the expression of K15 in odontogenic cysts and tumors. We have previously investigated the expression of K15 in dentigerous cyst, odontogenic keratocyst, and ameloblastoma (16). Moreover, the expression of COX-2 and K15 in RCs is poorly understood. Identifying the expression of these two markers may modify the current surgical treatment of RC such as targeting the inhibition of COX-2 enzyme. This study aimed at evaluating the expression of COX-2 and its relationship to K15 expression in the odontogenic epithelial cells of the RC.

## Material and Methods

In the current retrospective study, 18 RCs were included. The samples were retrieved from the Oral Pathology Department of Tongji Hospital. All samples had a definite diagnosis and an adequate epithelial component. The original H&E slides of all cases and their pathology reports were reviewed and confirmed. Baseline data including the patients’ age and gender as well as the location of the lesions were noted according to the patients’ medical files. This study followed the rules of the Declaration of Helsinki and was approved by the Institutional Review Board of Tongji Medical College. Informed consent was obtained from all subjects.

The size of the cyst was assessed by measuring the greatest cranio-caudal and mesio-distal diameters of the cyst on standard panoramic images and the arithmetic mean of those values was calculated as a final cyst size.

The immunostaining was done using the standard streptavidin‑biotin peroxidase complex (SABC) (Wuhan Boster Biological Technology, Ltd. China). After deparaffinization in xylene, the sections were dehydrated in alcohol and washed in distilled water. The activity of internal peroxidase was suppressed using 3% of hydrogen peroxide followed by the use of 0.01 M citrate buffer to unmask the antigen by heating in a microwave oven to the boiling point. Samples were treated with normal goat serum at room temperature for 50 min. Then, the slides were incubated with 1:100 diluted primary antibodies at 4 °C overnight. The used primary antibody for K15 immunostaining was mouse monoclonal antibody (BM0783; Clone: 6E7, Wuhan Boster Biological Technology, Ltd. China). Meanwhile, the primary antibody for COX-2 immunostaining was polyclonal rabbit anti‑human COX‑2 (BA0738; Wuhan Boster Biological Technology, Ltd. China). Later on, the samples were treated with appropriate biotinylated secondary antibodies (10 μg/ml) at room temperature for two hours. Subsequently, the sections were incubated with SABC (Wuhan Boster Biological Technology, Ltd. China). Finally, the sections were developed with a 3,3’-Diaminobenzidine substrate and counterstained with Mayer’s hematoxylin. For every experimental set, a sample was incubated with phosphate buffered saline (PBS) instead of the primary antibodies to serve as a negative control.

The cytoplasmic staining of K15 was yellowish to brown. Meanwhile, COX 2 expression was identified as different intensities of yellowish to brown staining that mainly located in the cytoplasm and nuclear membrane of the positive cells. Semi quantitative scoring under a light microscope was used. The scoring of K15 and COX-2 staining was measured by counting the percentage of positively stained cells from the total number of the cells in 10 high power representative fields, as follows: 0, when no identified staining or when the staining was questionable; mild for ≤ 25 % positivity; moderate for 26 %-50% positivity, and strong for >50 % positivity rate of the total epithelial cells.

The degree of chronic inflammation in the cystic capsule was classified as mild when the proportion of the chronic inflammatory cells was ≤ 25 % of the total cells; moderate for 26 %-50%, and strong for >50 %.

The statistical package for the social sciences (SPSS) 27.0 software was used for the statistical analyses. Correlations were analyzed using Spearman’s rank correlation coefficient test. Results were deemed statistically significant if *p*<0.05.

## Results

The study included 18 cases of RC, 12 in male patients and six in female patients, with a mean age of 36.50. There were 14 cases in the maxilla and four in the mandible. The samples consisted of relatively large size cysts where the mean size of the cysts was 3.0750 cm. Table 1 summarizes the most relevant clinical features.

**Table 1:**
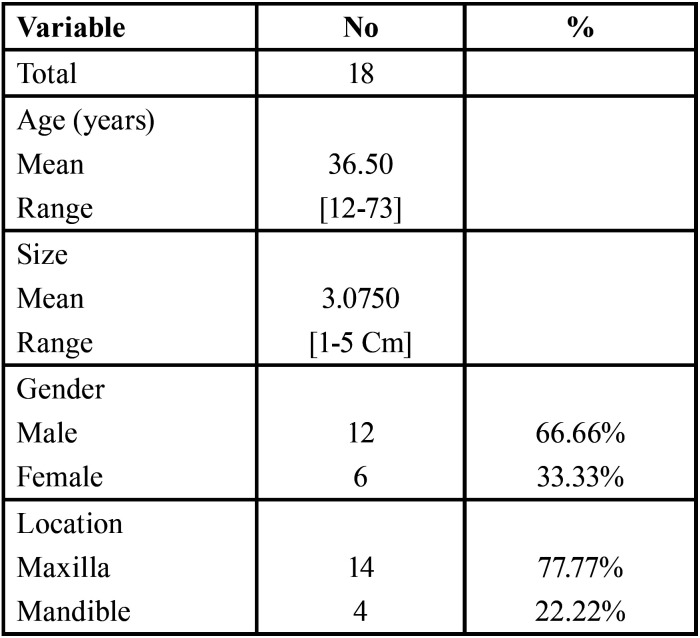
Clinical findings of the studied cases (n=18).

**Figure 1:**
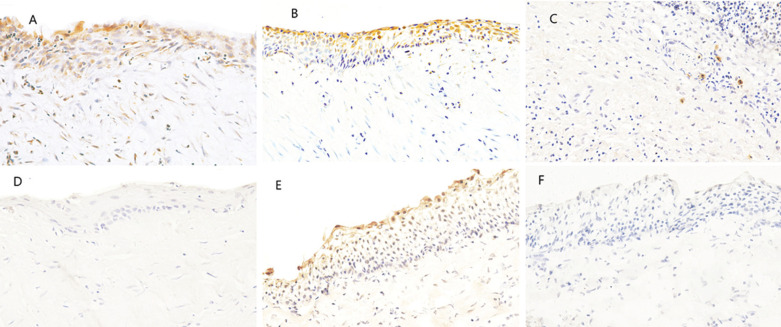
Immunohistochemical staining of cyclooxygenase-2 (COX-2) and Keratin 15 (K15) in radicular cysts (magnification, x 400). (a) COX-2 staining in the cytoplasm and nuclear membrane of the positive cells throughout the whole layers of the odontogenic epithelium. (b) COX-2 positivity is in the superior layers of epithelium in RC. (c) Positive COX-2 staining in the connective tissue capsule of different inflammatory cells. (d) Negative COX-2 staining in the epithelium and capsule. (e) K15 positive cytoplasmic staining in the upper layers of the odontogenic epithelium. (f) Negative K15 staining of the epithelium.

COX-2 staining was characterized by varying intensities of yellowish to brown staining in the cytoplasm and nuclear membrane of the odontogenic epithelium. The staining was distributed indiffirintly throughout the epithelium. However, some fields showed more staining in the superior layer of odontogenic epithelium (Fig. 1). In the connective tissue wall, COX-2 expression was observed in the inflammatory cells, endothelial cells, and fibroblasts (Fig. 1). COX-2 expression in the odontogenic epithelium of RC was absent in 11.1 % (n=2), mild in 27.8 % (n=5), moderate in 22.2% (n=4) and strong in 38.9% (n=7).

K15 expression was identified as yellowish to brown staining in the cytoplasm of the odontogenic epithelial cells of RC (Fig. 1). Positively stained cells were located mainly in the superior layer of the odontogenic epithelium (Fig. 1). K15 expression was absent in 27.8% of the cases (n=5), mild in 16.7% (n=3), moderate in 44.4% (n=8), and strong in 11.1% (n=2) (Table 2).

The inflammatory infiltrate was mild in 2 cases (11.1%), moderate in 6 cases (33.3%), and high in 10 cases (55.6%) (Table 2). Spearman’s correlation test showed significant correlation between COX-2 and K15 expressions in the odontogenic epithelium (rho= .533; *p*= .023). However, there was no significant correlations between chronic inflammatory reaction and COX-2 (rho= .248; *p*= .321) or K15 (rho= - .162; *p*= .520) expression (Table 3).

**Table 2:**
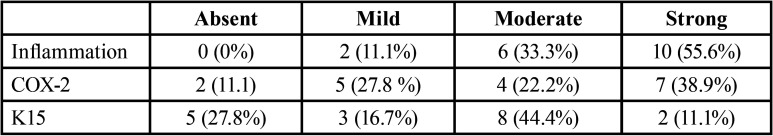
Degree of inflammation and expression of COX-2 and K15 in radicular cysts.

**Table 3:**
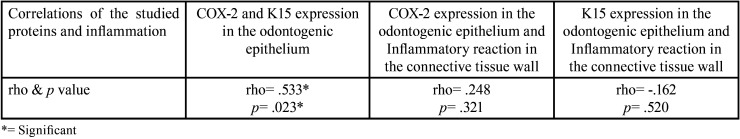
Spearman’s rank correlations between COX-2, K15, and chronic inflammatory reaction in radicular cysts.

## Discussion

RC is a pathological cavity that is completely or partly lined by epithelium in an area of apical periodontitis. A root canal infection of a tooth could result in the formation of a RC. The trigger and exact mechanism of RC formation is still not clear. However, it is believed that growth factors and inflammatory cytokines released in apical periodontitis can stimulate the remnant cells of epithelial rests of Malassez to proliferate and form a cyst.

The pathway of COX in periapical lesions has a wide modulation in genes expression involved in both bone anabolism and catabolism (17). In fact, resorption of the dense crystalline environment is the main requirement for a lesion to expand within the bone. As one possibility, it has been shown that COX-2 expression in the periapical lesion induces periapical bone resorption through prostaglandin E2 stimulation of osteoclastic bone resorption (4). Moreover, selective and nonselective COX-2 inhibitors have been found to regulate genes involved in bone metabolism (17). Furthermore, COX-2 upregulation leads to aggressive alveolar bone loss (18). The results of our study showed high expression of COX-2 in the odontogenic epithelium of the RC. It is conceivable that alveolar bone resorption is the main element proving the evolution from simple apical periodontitis to a reasonable sized RC. In addition, the high expression of COX-2 in the epithelium of RC may suggest a role for this enzyme in maintaining the survival and integrity of the cystic epithelium, which is usually not resolvable unless the cyst is surgically treated. In light of this, a previous study proposed an influential role for the intracellular location of the COX protein, where more COX-2 in the nuclear membrane could be associated with decreased apoptosis in epithelial cell lines (19). Hence, one could conclude a benefit of targeting COX-2 in the management of periapical lesions.

COX-2 expression was investigated in periapical granuloma and periodontitis (5,6), RC (5-7), odontogenic keratocyst (8,9), dentigerous cyst, and ameloblastoma (10). It has been found that RCs and periapical granulomas had a comparable expression of COX-2 (5,6). Moreover, it has been suggested that an important role of COX-2 may be played in the pathogenesis of RCs (5,7).

In the current study, the distribution of COX-2 expression was indifferent through all epithelial layers of RC. Additionally, COX-2 expression was detected in different cells of the cyst capsule such as the endothelial cells, fibroblasts, and inflammatory cells. These results are in agreement with previous reports that found a consistent expression of COX-2 in both the epithelium and connective tissue wall of RC (6,7).

Noteworthy, we found no correlation between epithelial COX-2 expression and the grade of inflammatory reaction in the connective tissue capsule of RC. This result is in agreement with a previous study that also did not reveal such a correlation (6). In contrast, Tsai *et al*. found that RCs with high levels of inflammation had significantly higher COX-2 expression as compared to RCs with low levels of inflammation (7). This controversy could be attributed to various methods used to calculate inflammatory infiltrate and COX-2 expression. The correlation between COX-2 expression and degree of inflammatory reaction was also investigated in different tissue types, where no such correlation was detected in gingivitis, periodontitis (20), or esophageal epithelium (21). Furthermore, Abdalla and coauthors suggested that COX-2 positivity is a response to non-inflammatory factors (21).

Several suggestions have been made regarding the expression of K15. It has been suggested that K15 is a stem cell marker based on early reports that found K15 expression occurs exclusively in the basal layers of skin surrounding the bulges of hair follicles. On the other hand, K15 was also shown to be an epithelial cell differentiation marker, based on the heavy expression and location in the superior epithelial layers of normal and pathological epithelial tissues. Specifically, K15 was used as a stem cell marker confirming its restricted expression to the bulge in hair follicles and the basal layer of stratified epithelia (22). Nonetheless, in some clinical conditions like oral lichen planus, K15 is expressed in suprabasal layers (23), which precludes its being an actual stem cell marker (24). Because of its huge and spatial distribution, a previous study excluded K15 being a stem cell marker in dentigerous cysts, odontogenic keratocysts, and ameloblastoma (16). Similarly, Porter *et al*. proposed that K15 was a marker of laterally differentiated keratinocytes and not a stem cell marker (25). Furthermore, in conditions of keratin diseases, the epithelial cell becomes fragile, lyse, and disaggregate under mild stress. Interestingly, Bose and coauthors proposed that K15 expression is regulated by two different mechanisms in stratified epithelia. One is basal-specific mediated by FOXM1 pathway and the other is differentiation-specific involving the PKC/AP-1 pathway (24).

To the extent of our knowledge, this is the first study that involved the investigation of K15 in RC. Detailed knowledge of K15 expression in RC could help in better understanding of the molecular pathogenesis and the subsequent non-surgical management of RC, the most common odontogenic cyst of the jaws. It is clear that different keratins are expressed in different types of epithelium. Moreover, keratins have specific locations of expression in different epithelial tissues. Regarding K15 expression in odontogenic lesions, there is only one previous study, conducted by our group, investigating K15 expression in dentigerous cyst, odontogenic keratocyst, and ameloblastoma (16). In the current study we found that K15 was expressed more in the upper layers of the epithelium than in the basal layer. This could indicate a more maturation and differentiation of the epithelial cells in these layers than in the basal layer. In line with this conclusion, K15 was found to be downregulated in hyperproliferating epidermis. The suppression of K15 expression could be attributed to the effect of growth factors and cytokines produced by activated and proliferating keratinocytes. Accordingly, a previous study found that proliferation was more prevalent in the basal layers than the superior layers of RC (26). Previously, we showed that K15 was distributed through the superior layers in dentigerous cyst and it was negatively associated with cellular proliferation (16).

It has been suggested that stress triggers a specific mechanism of differentiation through K15 expression in the stratified epithelia (24). This presumption could explain our finding that K15 is highly expressed in the odontogenic epithelium and mainly in the superior epithelial layers of RC, where more stress is anticipated. Alsaegh *et al*. concluded that the different location of K15 expression in dentigerous cyst and odontogenic keratocyst could be helpful in differentiating between these two lesions (16). Furthermore, the expression of keratin-7 was found in the superficial layers of the well-differentiated RC, but was significantly lower in the less differentiated odontogenic keratocysts, concluding that the existence of this keratin is correlated with the degree of differentiation of these cysts (27). The abundant expression and distribution of K15 in the studied samples outweighs the possibility of considering K15 as a marker of odontogenic epithelial cell differentiation rather than being a stem cells marker in RC.

The current study showed a negative, but not significant correlation between the expression of K15 and the degree of inflammation. The downregulation of K15 in inflammatory conditions is in agreement with the reported K15 suppression through the activation of specific cytokines and growth factors like tumor necrosis factor alpha, transforming growth factor beta, keratinocyte growth factor, and the epidermal growth factor (28). Indeed, during the early stages of RC formation, the epithelium may be more proliferative and associated with intense inflammatory process. However, when the RC enlarges, the lining becomes quiescent, regular, and more differentiated.

We found a significant positive correlation between COX-2 and K15 expressions in the epithelial lining of RC. Therefore, COX-2 could have a role in the induction of epithelial cells differentiation in RC. It appears that the effect of COX-2 on the odontogenic epithelium of RC is not dependent on the chronic inflammatory reaction in the cyst, as we did not find a correlation between COX-2 expression and the degree of the inflammatory reaction in the studied samples. A previous study demonstrated that COX-2 has a significant role in the regulation of epidermal cell differentiation, though the specific mechanism (s) that was not identified (29). Furthermore, the high concentrations of tumor necrosis factor inhibit epithelial cell proliferation (30). This means that excess tumor necrosis factor leads to less epithelial cell proliferation and eventually differentiation of the epithelial cells in the RC. In view of the close relation of tumor necrosis factor and COX-2 expressions, one could interpret the positive correlation between COX-2 and K15 by considering the effect of tumor necrosis factor on epithelial cells through the COX-2 pathway. However, further studies are recommended to prove this assumption. Taken together, COX-2 might be important for the differentiation and survival of the odontogenic epithelium of RC. Therefore, suppressing COX-2 expression, such as with NSAIDs, may be beneficial in the non-surgical treatment of RCs.

In conclusion, there is high and correlated expression of COX-2 and K15 in the odontogenic epithelium of RC. COX-2 could therefore be involved in epithelial cell differentiation of the cyst. Additionally, the expression of K15 in RC may be an indicator of epithelial cell differentiation. The results may lead to the future development of nonsurgical management of radicular cysts through the use of NSAIDs.
